# (*E*)-3-(4-Hexyl­oxyphen­yl)-1-(3-hydroxy­phen­yl)prop-2-en-one

**DOI:** 10.1107/S1600536809010617

**Published:** 2009-03-28

**Authors:** Zainab Ngaini, Siti Muhaini Haris Fadzillah, Norashikin Irdawaty Abd Rahman, Hasnain Hussain, Ibrahim Abdul Razak, Hoong-Kun Fun

**Affiliations:** a Department of Chemistry, Faculty of Resource Science and Technology, Universiti Malaysia Sarawak, 94300 Kota Samarahan, Sarawak, Malaysia; b Department of Molecular Biology, Faculty of Resource Science and Technology, Universiti Malaysia Sarawak, 94300 Kota Samarahan, Sarawak, Malaysia; c X-ray Crystallography Unit, School of Physics, Universiti Sains Malaysia, 11800 USM, Penang, Malaysia

## Abstract

In the title compound, C_21_H_24_O_3_, the enone unit is in the *s*–*cis* configuration. The dihedral angle between the benzene rings is 2.18 (4)°. In the crystal, mol­ecules are linked by pairs of O—H⋯O inter­molecular hydrogen bonds, forming inversion dimers. The crystal structure is also consolidated by C—H⋯π inter­actions.

## Related literature

For general background to the biological properties of chalcone derivatives, see: Bhat *et al.* (2005 ▶); Xue *et al.* (2004 ▶); Won *et al.* (2005 ▶); Yayli *et al.* (2006 ▶). For related structures, see: Ng, Razak *et al.* (2006 ▶); Ng, Patil *et al.* (2006 ▶). For details of hydrogen-bond motifs, see: Bernstein *et al.* (1995 ▶). For bond-length data, see: Allen *et al.* (1987 ▶). For the stability of the temperature controller uded in the data collection, see: Cosier & Glazer (1986 ▶).
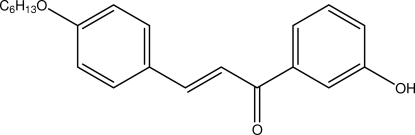


## Experimental

### 

#### Crystal data

C_21_H_24_O_3_
*M* *_r_* = 324.40
Monoclinic, 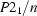

*a* = 8.5918 (2) Å
*b* = 17.1320 (3) Å
*c* = 12.4192 (2) Å
β = 109.083 (1)°
*V* = 1727.58 (6) Å^3^
*Z* = 4
Mo *K*α radiation
μ = 0.08 mm^−1^
*T* = 100 K
0.52 × 0.43 × 0.37 mm

#### Data collection

Bruker APEXII CCD area-detector diffractometer
Absorption correction: multi-scan (*SADABS*; Bruker, 2005 ▶) *T*_min_ = 0.959, *T*_max_ = 0.970
32772 measured reflections
7567 independent reflections
5739 reflections with *I* > 2σ(*I*)
*R*_int_ = 0.026

#### Refinement

*R*[*F* ^2^ > 2σ(*F* ^2^)] = 0.043
*wR*(*F* ^2^) = 0.131
*S* = 1.04
7567 reflections
222 parameters
H atoms treated by a mixture of independent and constrained refinement
Δρ_max_ = 0.48 e Å^−3^
Δρ_min_ = −0.22 e Å^−3^

### 

Data collection: *APEX2* (Bruker, 2005 ▶); cell refinement: *SAINT* (Bruker, 2005 ▶); data reduction: *SAINT*; program(s) used to solve structure: *SHELXTL* (Sheldrick, 2008 ▶); program(s) used to refine structure: *SHELXTL*; molecular graphics: *SHELXTL*; software used to prepare material for publication: *SHELXTL* and *PLATON* (Spek, 2009 ▶).

## Supplementary Material

Crystal structure: contains datablocks global, I. DOI: 10.1107/S1600536809010617/fj2200sup1.cif

Structure factors: contains datablocks I. DOI: 10.1107/S1600536809010617/fj2200Isup2.hkl

Additional supplementary materials:  crystallographic information; 3D view; checkCIF report

## Figures and Tables

**Table 1:** Hydrogen-bond geometry (Å, °)

*D*—H⋯*A*	*D*—H	H⋯*A*	*D*⋯*A*	*D*—H⋯*A*
O1—H101⋯O2^i^	0.86 (2)	1.89 (2)	2.739 (1)	171 (2)
C16—H16*A*⋯*Cg*1^ii^	0.97	2.72	3.572 (1)	146
C20—H20*A*⋯*Cg*1^iii^	0.97	2.82	3.642 (1)	143

Symmetry codes: (i) 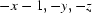; (ii) 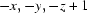; (iii) 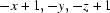. *Cg*1 is the centroid of C1–C6 ring.

**Fig. 1.**
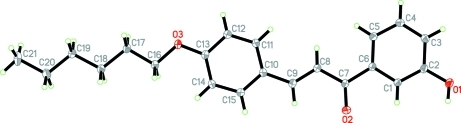
The asymmetric unit of the title compound, showing 50% probability displacement ellipsoids and the atom numbering scheme. Intramolecular H-bonds are drawn as dashed lines.

**Fig. 2.**
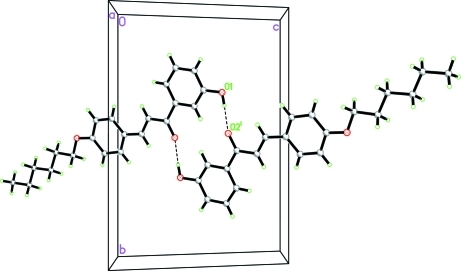
The packing viewed down the a axis showing the dimer formation. The symmetry code is given in Table 2.

## supplementary crystallographic information

### Comment

Chalcone is a common natural pigment and one of the important intermediate in the biosynthesis of flavonoid. Synthetic and naturally occurring chalcones have been extensively studied and developed as one of the pharmaceutically important molecules. Chalcone derivatives are reported to possess a broad spectrum of biological properties such as an anticancer (Bhat *et al.*, 2005) antimalarial (Xue *et al.*, 2004), anti-inflammatory (Won *et al.*, 2005), and antioxidant and antimicrobial activities (Yayli *et al.*, 2006).

The synthesis of chalcone derivatives possessing alkyl chains of varying length has been synthesized in our lab and their antibacterial activities was tested against *E. coli* ATCC 8739. All the synthesized chalcone derivatives showed antimicrobial activity. In this paper, we report the structure of the title compound which is one of the chalcone derivatives mentioned above.

The bond lengths (Allen *et al.*, 1987) and angles observed in (I) show normal values. The least-square plane through the enone moiety (O2C7C8C9) makes dihedral angles of 5.32 (5)° and 4.72 (5)° with the C1—C6 and C10—C15 benzene rings, respectively. The dihedral angle between these benzene rings is 2.18 (4)°. The alkoxyl tail is coplanar with the attached ring with the torsion angle C16—O3—C13—C14 being -0.26 (11)°.

The O2—C7—C8—C9 torsion angle of 4.1 (1)° shows that the enone moiety is in the s-*cis* configuration. The short H5A···H8A (2.12 Å) contact results in the widening of C5—C6—C7 (123.22 (7)°) angle while the widening of C8—C9—C10 (128.33 (7)°) and C9—C10—C11 (124.01 (7)°) angles are the result of close H8A···H11A (2.32 Å) contact. Similar strain induced by short H14A···H16A (2.32 Å) and H14A···H16A (2.28 Å) has also widened the C14—C13—O3 (124.19 (7)°) angles. These observations are also mentioned in structures reported by Ng, Razak *et al.* (2006) and Ng, Patil *et al.* (2006).

In the crystal, O1-H1O1···O2(-*x* - 1,-*y*,-*z*) intermolecular hydrogen bonds involving the keto and the hydroxy O atoms form molecular dimers. The crystal structure is further stabilized by C—H···π interactions.

### Experimental

A mixture of 3-hydroxyacetophenone (1.23 g, 9 mmol) and 4-hexyloxybenzaldehyde (1.86 ml, 9 mmol) and KOH (1.82 g, 32.4 mmol) in 30 ml of methanol was heated at reflux for 12 h. The reaction was cooled to room temperature and acidified with cold diluted HCl (2 N). The resulting precipitate was filtered, washed and dried. The precipitate was dissolved in hexane–ethanol (7:1) mixture. After a few days of slow evaporation, colourless crystals were collected for X-ray analysis.

### Refinement

All the H atoms were positioned geometrically and refined using a riding model with C—H = 0.93–0.97 Å. The *U*_iso_ values were constrained to be -1.5U_equ_ (methyl H atoms) and -1.2U_equ_ (other H atoms). The rotating model group was considered for the methyl group. In the case of O1, the hydrogen atom was located from a difference Fourier map and refined isotropically.

### Crystal data

**Table:**

C_21_H_24_O_3_	*F*(000) = 696
*M**_r_* = 324.40	*D*_x_ = 1.247 Mg m^−^^3^
Monoclinic, *P*2_1_/*n*	Mo *K*α radiation, λ = 0.71073 Å
Hall symbol: -P 2yn	Cell parameters from 9979 reflections
*a* = 8.5918 (2) Å	θ = 2.8–39.2°
*b* = 17.1320 (3) Å	µ = 0.08 mm^−^^1^
*c* = 12.4192 (2) Å	*T* = 100 K
β = 109.083 (1)°	Block, colourless
*V* = 1727.58 (6) Å^3^	0.52 × 0.43 × 0.37 mm
*Z* = 4	

### Data collection

**Table:**

Bruker APEXII CCD area-detector diffractometer	7567 independent reflections
Radiation source: sealed tube	5739 reflections with *I* > 2σ(*I*)
graphite	*R*_int_ = 0.026
φ and ω scans	θ_max_ = 35.0°, θ_min_ = 2.1°
Absorption correction: multi-scan (*SADABS*; Bruker, 2005)	*h* = −13→13
*T*_min_ = 0.959, *T*_max_ = 0.970	*k* = −22→27
32772 measured reflections	*l* = −18→20

### Refinement

**Table:**

Refinement on *F*^2^	Primary atom site location: structure-invariant direct methods
Least-squares matrix: full	Secondary atom site location: difference Fourier map
*R*[*F*^2^ > 2σ(*F*^2^)] = 0.043	Hydrogen site location: inferred from neighbouring sites
*wR*(*F*^2^) = 0.131	H atoms treated by a mixture of independent and constrained refinement
*S* = 1.04	*w* = 1/[σ^2^(*F*_o_^2^) + (0.0658*P*)^2^ + 0.3447*P*] where *P* = (*F*_o_^2^ + 2*F*_c_^2^)/3
7567 reflections	(Δ/σ)_max_ = 0.001
222 parameters	Δρ_max_ = 0.48 e Å^−^^3^
0 restraints	Δρ_min_ = −0.22 e Å^−^^3^

### Special details

**Table:**

Experimental. The crystal was placed in the cold stream of an Oxford Cryosystems Cobra open-flow nitrogen cryostat (Cosier & Glazer, 1986) operating at 100.0 (1) K.
Geometry. All e.s.d.'s (except the e.s.d. in the dihedral angle between two l.s. planes) are estimated using the full covariance matrix. The cell e.s.d.'s are taken into account individually in the estimation of e.s.d.'s in distances, angles and torsion angles; correlations between e.s.d.'s in cell parameters are only used when they are defined by crystal symmetry. An approximate (isotropic) treatment of cell e.s.d.'s is used for estimating e.s.d.'s involving l.s. planes.
Refinement. Refinement of *F*^2^ against ALL reflections. The weighted *R*-factor *wR* and goodness of fit *S* are based on *F*^2^, conventional *R*-factors *R* are based on *F*, with *F* set to zero for negative *F*^2^. The threshold expression of *F*^2^ > σ(*F*^2^) is used only for calculating *R*-factors(gt) *etc*. and is not relevant to the choice of reflections for refinement. *R*-factors based on *F*^2^ are statistically about twice as large as those based on *F*, and *R*- factors based on ALL data will be even larger.

### Fractional atomic coordinates and isotropic or equivalent isotropic displacement parameters (Å^2^)

**Table:**

	*x*	*y*	*z*	*U*_iso_*/*U*_eq_	
O1	−0.67033 (8)	−0.16211 (4)	−0.11538 (5)	0.02019 (13)	
O2	−0.24091 (8)	0.00815 (4)	0.14823 (5)	0.01889 (12)	
O3	0.57829 (7)	0.01121 (4)	0.74453 (5)	0.01808 (12)	
C1	−0.43937 (9)	−0.11205 (4)	0.03753 (6)	0.01426 (13)	
H1A	−0.4490	−0.0636	0.0023	0.017*	
C2	−0.54671 (10)	−0.17174 (5)	−0.01437 (6)	0.01515 (14)	
C3	−0.53013 (10)	−0.24523 (5)	0.03709 (7)	0.01738 (15)	
H3A	−0.6009	−0.2856	0.0020	0.021*	
C4	−0.40736 (10)	−0.25769 (5)	0.14089 (7)	0.01789 (15)	
H4A	−0.3966	−0.3066	0.1751	0.021*	
C5	−0.30007 (10)	−0.19778 (5)	0.19442 (7)	0.01605 (14)	
H5A	−0.2183	−0.2066	0.2639	0.019*	
C6	−0.31644 (9)	−0.12429 (4)	0.14274 (6)	0.01352 (13)	
C7	−0.20986 (9)	−0.05615 (5)	0.19509 (6)	0.01364 (13)	
C8	−0.06986 (10)	−0.06665 (5)	0.30060 (6)	0.01473 (13)	
H8A	−0.0520	−0.1146	0.3376	0.018*	
C9	0.03228 (10)	−0.00634 (5)	0.34327 (6)	0.01478 (14)	
H9A	0.0084	0.0398	0.3016	0.018*	
C10	0.17496 (9)	−0.00406 (5)	0.44610 (6)	0.01412 (13)	
C11	0.22794 (10)	−0.06753 (5)	0.52147 (7)	0.01577 (14)	
H11A	0.1714	−0.1147	0.5049	0.019*	
C12	0.36209 (10)	−0.06102 (5)	0.61936 (7)	0.01651 (14)	
H12A	0.3952	−0.1036	0.6679	0.020*	
C13	0.44893 (9)	0.00985 (5)	0.64597 (6)	0.01485 (14)	
C14	0.39946 (10)	0.07342 (5)	0.57204 (7)	0.01645 (14)	
H14A	0.4560	0.1205	0.5886	0.020*	
C15	0.26459 (10)	0.06536 (5)	0.47334 (7)	0.01590 (14)	
H15A	0.2331	0.1076	0.4239	0.019*	
C16	0.66971 (10)	0.08312 (5)	0.77343 (7)	0.01598 (14)	
H16A	0.5981	0.1248	0.7818	0.019*	
H16B	0.7137	0.0975	0.7135	0.019*	
C17	0.80851 (10)	0.07101 (5)	0.88414 (7)	0.01625 (14)	
H17A	0.8812	0.0303	0.8742	0.019*	
H17B	0.7636	0.0540	0.9425	0.019*	
C18	0.90687 (10)	0.14613 (5)	0.92278 (7)	0.01651 (14)	
H18A	0.9536	0.1620	0.8649	0.020*	
H18B	0.8326	0.1871	0.9295	0.020*	
C19	1.04510 (10)	0.13787 (5)	1.03599 (7)	0.01726 (14)	
H19A	1.1188	0.0966	1.0296	0.021*	
H19B	0.9984	0.1227	1.0942	0.021*	
C20	1.14369 (11)	0.21270 (5)	1.07309 (8)	0.02270 (17)	
H20A	1.1893	0.2283	1.0145	0.027*	
H20B	1.0704	0.2538	1.0806	0.027*	
C21	1.28308 (13)	0.20366 (7)	1.18553 (9)	0.0326 (2)	
H21A	1.3478	0.2505	1.2015	0.049*	
H21B	1.3514	0.1604	1.1805	0.049*	
H21C	1.2379	0.1943	1.2456	0.049*	
H101	−0.6888 (18)	−0.1130 (9)	−0.1279 (12)	0.042 (4)*	

### Atomic displacement parameters (Å^2^)

**Table:**

	*U*^11^	*U*^22^	*U*^33^	*U*^12^	*U*^13^	*U*^23^
O1	0.0200 (3)	0.0164 (3)	0.0173 (3)	0.0002 (2)	−0.0033 (2)	−0.0017 (2)
O2	0.0204 (3)	0.0150 (3)	0.0177 (3)	−0.0006 (2)	0.0015 (2)	0.0025 (2)
O3	0.0152 (3)	0.0188 (3)	0.0162 (3)	−0.0034 (2)	−0.0005 (2)	−0.0007 (2)
C1	0.0138 (3)	0.0135 (3)	0.0142 (3)	0.0008 (2)	0.0029 (3)	0.0001 (2)
C2	0.0138 (3)	0.0162 (3)	0.0137 (3)	0.0009 (3)	0.0020 (2)	−0.0016 (2)
C3	0.0171 (3)	0.0145 (3)	0.0187 (3)	−0.0014 (3)	0.0033 (3)	−0.0015 (3)
C4	0.0192 (4)	0.0138 (3)	0.0190 (3)	0.0003 (3)	0.0038 (3)	0.0017 (3)
C5	0.0165 (3)	0.0155 (3)	0.0144 (3)	0.0008 (3)	0.0026 (3)	0.0010 (3)
C6	0.0125 (3)	0.0143 (3)	0.0131 (3)	0.0005 (2)	0.0034 (2)	−0.0006 (2)
C7	0.0129 (3)	0.0151 (3)	0.0127 (3)	0.0001 (2)	0.0037 (2)	−0.0003 (2)
C8	0.0134 (3)	0.0156 (3)	0.0137 (3)	0.0003 (2)	0.0025 (2)	0.0006 (2)
C9	0.0136 (3)	0.0161 (3)	0.0139 (3)	0.0004 (3)	0.0036 (2)	−0.0008 (2)
C10	0.0128 (3)	0.0156 (3)	0.0136 (3)	−0.0009 (2)	0.0039 (2)	−0.0013 (2)
C11	0.0146 (3)	0.0153 (3)	0.0164 (3)	−0.0019 (3)	0.0037 (3)	−0.0007 (3)
C12	0.0155 (3)	0.0164 (3)	0.0161 (3)	−0.0008 (3)	0.0030 (3)	0.0009 (3)
C13	0.0123 (3)	0.0179 (3)	0.0136 (3)	−0.0004 (3)	0.0032 (2)	−0.0014 (2)
C14	0.0157 (3)	0.0156 (3)	0.0168 (3)	−0.0025 (3)	0.0035 (3)	−0.0013 (3)
C15	0.0160 (3)	0.0148 (3)	0.0156 (3)	−0.0006 (3)	0.0033 (3)	0.0004 (3)
C16	0.0135 (3)	0.0175 (3)	0.0160 (3)	−0.0021 (3)	0.0035 (3)	−0.0019 (3)
C17	0.0133 (3)	0.0190 (3)	0.0150 (3)	−0.0011 (3)	0.0025 (3)	−0.0004 (3)
C18	0.0143 (3)	0.0172 (3)	0.0161 (3)	−0.0003 (3)	0.0023 (3)	−0.0003 (3)
C19	0.0152 (3)	0.0190 (4)	0.0153 (3)	−0.0007 (3)	0.0020 (3)	−0.0003 (3)
C20	0.0204 (4)	0.0199 (4)	0.0235 (4)	−0.0004 (3)	0.0012 (3)	−0.0051 (3)
C21	0.0271 (5)	0.0418 (6)	0.0223 (4)	−0.0072 (4)	−0.0010 (4)	−0.0104 (4)

### Geometric parameters (Å, °)

**Table:**

O1—C2	1.3631 (9)	C12—C13	1.4067 (11)
O1—H101	0.861 (16)	C12—H12A	0.9300
O2—C7	1.2336 (9)	C13—C14	1.3980 (11)
O3—C13	1.3581 (9)	C14—C15	1.3912 (11)
O3—C16	1.4419 (10)	C14—H14A	0.9300
C1—C2	1.3867 (11)	C15—H15A	0.9300
C1—C6	1.4016 (11)	C16—C17	1.5111 (11)
C1—H1A	0.9300	C16—H16A	0.9700
C2—C3	1.3980 (11)	C16—H16B	0.9700
C3—C4	1.3895 (11)	C17—C18	1.5282 (11)
C3—H3A	0.9300	C17—H17A	0.9700
C4—C5	1.3944 (11)	C17—H17B	0.9700
C4—H4A	0.9300	C18—C19	1.5217 (11)
C5—C6	1.3993 (11)	C18—H18A	0.9700
C5—H5A	0.9300	C18—H18B	0.9700
C6—C7	1.4949 (11)	C19—C20	1.5225 (12)
C7—C8	1.4717 (11)	C19—H19A	0.9700
C8—C9	1.3470 (11)	C19—H19B	0.9700
C8—H8A	0.9300	C20—C21	1.5214 (13)
C9—C10	1.4531 (11)	C20—H20A	0.9700
C9—H9A	0.9300	C20—H20B	0.9700
C10—C15	1.3971 (11)	C21—H21A	0.9600
C10—C11	1.4096 (11)	C21—H21B	0.9600
C11—C12	1.3794 (11)	C21—H21C	0.9600
C11—H11A	0.9300		
			
C2—O1—H101	109.0 (10)	C15—C14—H14A	120.4
C13—O3—C16	117.24 (6)	C13—C14—H14A	120.4
C2—C1—C6	120.45 (7)	C14—C15—C10	122.15 (7)
C2—C1—H1A	119.8	C14—C15—H15A	118.9
C6—C1—H1A	119.8	C10—C15—H15A	118.9
O1—C2—C1	122.56 (7)	O3—C16—C17	108.23 (6)
O1—C2—C3	117.51 (7)	O3—C16—H16A	110.1
C1—C2—C3	119.92 (7)	C17—C16—H16A	110.1
C4—C3—C2	119.70 (7)	O3—C16—H16B	110.1
C4—C3—H3A	120.1	C17—C16—H16B	110.1
C2—C3—H3A	120.1	H16A—C16—H16B	108.4
C3—C4—C5	120.81 (7)	C16—C17—C18	111.16 (7)
C3—C4—H4A	119.6	C16—C17—H17A	109.4
C5—C4—H4A	119.6	C18—C17—H17A	109.4
C4—C5—C6	119.47 (7)	C16—C17—H17B	109.4
C4—C5—H5A	120.3	C18—C17—H17B	109.4
C6—C5—H5A	120.3	H17A—C17—H17B	108.0
C5—C6—C1	119.63 (7)	C19—C18—C17	113.34 (7)
C5—C6—C7	123.22 (7)	C19—C18—H18A	108.9
C1—C6—C7	117.14 (7)	C17—C18—H18A	108.9
O2—C7—C8	121.13 (7)	C19—C18—H18B	108.9
O2—C7—C6	119.01 (7)	C17—C18—H18B	108.9
C8—C7—C6	119.86 (7)	H18A—C18—H18B	107.7
C9—C8—C7	119.62 (7)	C18—C19—C20	112.97 (7)
C9—C8—H8A	120.2	C18—C19—H19A	109.0
C7—C8—H8A	120.2	C20—C19—H19A	109.0
C8—C9—C10	128.33 (7)	C18—C19—H19B	109.0
C8—C9—H9A	115.8	C20—C19—H19B	109.0
C10—C9—H9A	115.8	H19A—C19—H19B	107.8
C15—C10—C11	117.59 (7)	C21—C20—C19	112.66 (8)
C15—C10—C9	118.39 (7)	C21—C20—H20A	109.1
C11—C10—C9	124.01 (7)	C19—C20—H20A	109.1
C12—C11—C10	121.22 (7)	C21—C20—H20B	109.1
C12—C11—H11A	119.4	C19—C20—H20B	109.1
C10—C11—H11A	119.4	H20A—C20—H20B	107.8
C11—C12—C13	120.19 (7)	C20—C21—H21A	109.5
C11—C12—H12A	119.9	C20—C21—H21B	109.5
C13—C12—H12A	119.9	H21A—C21—H21B	109.5
O3—C13—C14	124.19 (7)	C20—C21—H21C	109.5
O3—C13—C12	116.20 (7)	H21A—C21—H21C	109.5
C14—C13—C12	119.61 (7)	H21B—C21—H21C	109.5
C15—C14—C13	119.22 (7)		
			
C6—C1—C2—O1	178.55 (7)	C8—C9—C10—C11	−1.52 (13)
C6—C1—C2—C3	−1.49 (11)	C15—C10—C11—C12	0.84 (11)
O1—C2—C3—C4	−179.16 (7)	C9—C10—C11—C12	−178.31 (7)
C1—C2—C3—C4	0.88 (12)	C10—C11—C12—C13	0.14 (12)
C2—C3—C4—C5	−0.11 (12)	C16—O3—C13—C14	−0.26 (11)
C3—C4—C5—C6	−0.05 (12)	C16—O3—C13—C12	179.73 (7)
C4—C5—C6—C1	−0.54 (11)	C11—C12—C13—O3	179.37 (7)
C4—C5—C6—C7	178.46 (7)	C11—C12—C13—C14	−0.64 (12)
C2—C1—C6—C5	1.32 (11)	O3—C13—C14—C15	−179.87 (7)
C2—C1—C6—C7	−177.75 (7)	C12—C13—C14—C15	0.13 (12)
C5—C6—C7—O2	−174.30 (7)	C13—C14—C15—C10	0.89 (12)
C1—C6—C7—O2	4.73 (10)	C11—C10—C15—C14	−1.37 (11)
C5—C6—C7—C8	6.05 (11)	C9—C10—C15—C14	177.83 (7)
C1—C6—C7—C8	−174.92 (7)	C13—O3—C16—C17	−178.97 (6)
O2—C7—C8—C9	−4.05 (11)	O3—C16—C17—C18	−177.65 (6)
C6—C7—C8—C9	175.59 (7)	C16—C17—C18—C19	178.18 (6)
C7—C8—C9—C10	179.11 (7)	C17—C18—C19—C20	179.31 (7)
C8—C9—C10—C15	179.34 (8)	C18—C19—C20—C21	−179.22 (8)

### Hydrogen-bond geometry (Å, °)

**Table:**

*D*—H···*A*	*D*—H	H···*A*	*D*···*A*	*D*—H···*A*
O1—H101···O2^i^	0.86 (2)	1.89 (2)	2.739 (1)	171 (2)
C16—H16A···Cg1^ii^	0.97	2.72	3.572 (1)	146
C20—H20A···Cg1^iii^	0.97	2.82	3.642 (1)	143

Symmetry codes: (i) −*x*−1, −*y*, −*z*; (ii) −*x*, −*y*, −*z*+1; (iii) −*x*+1, −*y*, −*z*+1.
